# Kinome-Wide siRNA Screening Identifies DYRK1B as a Potential Therapeutic Target for Triple-Negative Breast Cancer Cells

**DOI:** 10.3390/cancers13225779

**Published:** 2021-11-18

**Authors:** Chia-Che Chang, Chien-Chih Chiu, Pei-Feng Liu, Chih-Hsuan Wu, Yen-Chiang Tseng, Cheng-Hsin Lee, Chih-Wen Shu

**Affiliations:** 1 Department of Oncology, Zuoying Branch of Kaohsiung Armed Forces General Hospital, Kaohsiung 81300, Taiwan; Jeffisdog1980@gmail.com; 2 Department of Biotechnology, Kaohsiung Medical University, Kaohsiung 80708, Taiwan; cchiu@kmu.edu.tw; 3 Department of Biomedical Science and Environmental Biology, PhD Program in Life Science, College of Life Science, Kaohsiung Medical University, Kaohsiung 80708, Taiwan; pfliu@kmu.edu.tw (P.-F.L.); angioadsc@gmail.com (C.-H.L.); 4 Department of Medical Research, Kaohsiung Medical University Hospital, Kaohsiung 80708, Taiwan; pinktamago@gmail.com; 5 Center for Cancer Research, Kaohsiung Medical University, Kaohsiung 80708, Taiwan; 6 Department of Surgery, Division of Thoracic Surgery, Kaohsiung Veterans General Hospital, Kaohsiung 81362, Taiwan; yctseng@vghks.gov.tw; 7 Institute of Clinical Medicine, National Yang-Ming University, Taipei 11221, Taiwan; 8 Institute of BioPharmaceutical Sciences, National Sun Yat-sen University, No. 70, Lianhai Rd., Gushan Dist., Kaohsiung 80424, Taiwan

**Keywords:** kinome, siRNA, screening, DYRK1B, prognosis, triple-negative breast cancer

## Abstract

**Simple Summary:**

Therapeutic target is limited for patients with triple-negative breast cancer (TNBC). Through kinome-wide siRNA (709 genes) screening, DYRK1B was identified as a potential gene essential for cell proliferation and mobility of TNBC cells, particularly in DYRK1B highly expressed TNBC cells. TNBC patients with high expression of DYRK1B had poor overall survival and disease-free survival. CCDC97 and ZNF581 were positively correlated with DYRK1B expression and might be involved in DYRK1B-mediated tumor malignancy in TNBC patients, providing DYRK1B as a potential theranostic target for TNBC.

**Abstract:**

Aims: The selective molecules for targeted therapy of triple-negative breast cancer (TNBC) are limited. Several kinases play pivotal roles in cancer development and malignancy. The study aims to determine if any kinases confer to malignancy of TNBC cells, which could serve as a theranostic target for TNBC. Methods: Kinome siRNA library was used to screen selective genes required for the proliferation of TNBC cells. The involvement of DYRK1B in cancer malignancy was evaluated with migration, invasion assays, and spheroid culture. The expression of DYRK1B was confirmed with quantitative PCR and immunoblotting. The clinical correlation of DYRK1B in TNBC patients was examined with tissue microarray and The Cancer Genome Atlas (TCGA) database. Results: Our results showed that silencing DYRK1B significantly suppressed cell viability in DYRK1B-high expressed TNBC cells, likely by arresting the cell cycle at the G_1_ phase. Nevertheless, silencing DYRK1B had marginal effects on DYRK1B-low expressed TNBC cells. Similarly, the knockdown of DYRK1B decreased tumorsphere formation and increased cell death of the tumorsphere. Moreover, inactivation of DYRK1B by either specific inhibitor or ectopic expressing catalytic mutant of DYRK1B inhibited cell viability and metastatic characteristics, including migration and invasion. In addition, DYRK1B protein expression was elevated in tumor tissues compared to that in adjacent normal tissues of TNBC patients. Further, DYRK1B gene expression was highly correlated with CCDC97 or ZNF581 genes in TNBC cells and patients. High co-expression of DYRK1B with CCDC97 or ZNF581 was significantly associated with unfavorable overall survival and disease-free survival of TNBC patients. Conclusions: our results suggest DYRK1B might be essential for promoting tumor progression and could be a theranostic target for TNBC. Silencing or inactivation of DYRK1B might be a potential targeted therapy for TNBC.

## 1. Introduction

Breast cancer is the most common type of cancer among women and is a leading cause of cancer death worldwide [1]. Human epidermal growth factor receptor 2 (HER2) estrogen (ER) and progesterone (PgR) are three cell surface molecules typically used as therapeutic targets in current breast cancer treatment. Triple-negative breast cancer (TNBC) is defined as a tumor with no or little expression for these three surface molecules, thus presently has limited targeted therapy [2]. TNBC patients currently treated with conventional chemotherapy, including anthracycline and taxane, do not exhibit a striking increase in pathological complete response (pCR), ranging 30–40% for early stage TNBC patients [1]. Though several biomarkers have been reported as diagnostic and prognostic candidates [3,4], a therapeutic target is still an urgent need for TNBC patients.

About 538 kinases are encoded in the human kinome, including 89 tyrosine kinases, 429 serine/threonine kinases, and 20 lipid kinases. Kinases play crucial roles in signal transduction for a variety of cell physiological functions, such as cell proliferation, migration, and differentiation. Some kinases, particularly tyrosine kinases, are involved in tumorigenesis, malignancy, and drug resistance. These kinases have been used as targets for targeted cancer therapy in clinical settings. For example, Gleevec blocks BCR-Abl fusion kinase to treat CML [5], and Cetuximab inhibits EGFR signaling to treat lung cancer [6], and colorectal cancer (Ras wild type) [7]. Trastuzumab is the humanized monoclonal antibody targeting HER2 and extends overall survival of HER2-positive breast cancer patients compared to that in HER2-negative breast cancer patients [8]. These targeted cancer therapies successfully suppress tumor growth and malignancy in clinical settings. Regarding the role of kinases in TNBC, frequent mutation of phosphoinositide 3 (PI3)-kinase (PIK3CA) and AKT1 (also known as protein kinase B) have been found in TNBC patients [9]. Mutation of PI3KCA and AKT1 results in highly activation of the kinases and their downstream effector mammalian target of rapamycin (mTOR). Targeting the kinase causes feedback loop to reactivate the kinase activity and limits efficacy of kinase inhibitors [10]. Dual blockage of these kinases causes severe side effects in clinical trials [11]. Thus, it is still not clear which kinase could be a theranostic target for TNBC. 

Functional genomic screening with a small interfering RNA (siRNA) library provides a formidable tool to identify either essential genes or drug-resistant genes in different cancer cells as potential therapeutic targets. For example, kinome-wide siRNA screening results indicate that polo-like kinase 1 (PLK-1) is a crucial gene for cell viability in oral and colorectal cancer cells [12,13]. Meanwhile, Mitogen-activated protein kinase 7 (MAP3K7) is required for cell proliferation in hepatocellular carcinoma [14]. The expression of these genes is also increased in tumor tissues and linked to poor survival in cancer patients [12,14]. Moreover, siRNA screening reveals that several genes, such as PTEN-induced kinase 1 (PINK1) and Cysteine rich motor neuron 1 (CRIM1), may play drug-resistance roles in colorectal cancer cells when exposed to MEK inhibitor trametinib [13]. Knockdown of Src confers to resistance to doxorubicin, possibly through Protein kinase B (AKT) activation and expression of the signal transducer and activator of transcription 3 (STAT3) in TNBC cell lines [15]. These studies suggest that kinome siRNA library screening could be applied to identify suitable therapeutic targets for TNBC.

In this study, the kinome siRNA library was used to screen essential genes required for TNBC cell survival using normal breast cells as a counter assay. Silencing of the kinase hits was validated in four different TNBC cells and their effects in cell cycle progression, migration, invasion, and tumorsphere formation were examined. The expression of the candidate gene dual specificity tyrosine phosphorylation regulated kinase 1B (DYRK1B) was further analyzed for correlation with clinicopathological outcomes using The Cancer Genome Atlas (TCGA) dataset. Through whole genome data analysis in TCGA database, the expression of ZNF581 and CCDC97 was strongly positive correlated with DYRK1B in TNBC patients. The correlation of genes expression was also consistent in TNBC cells. The co-expression of DYRK1B/ZNF581 or CCDC97 genes with overall survival and disease-free survival of TNBC patients were also analyzed. Our results may provide a platform to identify potential targets for cancer therapy, and, hopefully, present DYRK1B as therapeutic target for TNBC. 

## 2. Materials and Methods

### 2.1. Cell Culture and Transfection

Human TNBC cell lines H184B5F5/M10, MDA-MB-231, MDA-MB-468, HCC1937, and Hs578T were purchased from American Type Culture Collection (ATCC). Highly invasive MDA-MB-231-IV2-1 cells were kindly provided by Dr. Lu-Hai Wang [16]. All cells were cultured as previously described [15]. For kinase gene screening, 10 nM arrayed siRNA against kinase genes (2127 siRNA, 709 genes, A30079, Thermo Fisher Scientific, Carlsbad, CA, USA) were spotted into each well of a 384-well plate along with lipofectamine RNAiMAX (Thermo Fisher Scientific, 13778-150). The TNBC cells were seeded in the plate containing siRNA for 72 h to further measure cell proliferation, as described previously [12,14]. To transiently knock down genes with siRNA, cells were transfected with 10 nM scramble siRNA (4390843, Thermo Fisher Scientific or D-001810-10-05, Dharmacon, CO, USA) or pooled siRNA against DYRK1B kinase (#1: s17489, #2: s17490, #3: s17491, Thermo Fisher Scientific) or CCDC97/ZNF581 D-(001810-10-05, Dharmacon, CO, USA) in the presence of lipofectamine RNAiMAX. To generate the shRNA stable cell line, off-targeting shRNA and shRNAs against DYRK1B (TRCN0000002139) obtained from The RNAi Consortium (TRC, Taiwan) were transfected into 293FT cells for 2 d, and the supernatant was used to infect TNBC cells. For transient transfection with expression vector encoding DYRK1B wild-type (addgene #17846) and catalytic mutant (addgene #17845), 2 µg plasmid were mixed with Lipofectamine 3000 (Thermo Fisher Scientific, L300008) and transfected into breast cancer cells.

### 2.2. Cell Viability Assays

To assay cell viability in kinome siRNA screening, 10 nM scramble siRNA or kinome siRNA library was mixed with lipofectamine RNAiMAX in 384-well white plates for more than 10 min. The TNBC cells (2 × 10^3^/well) were seeded for 72 h and then mixed with Cell-Titer Glo (G7572, Promega) for 10 min to lyse and measure cellular ATP level. The luminescent signal read by a Fluoroskan Ascent FL reader (Thermo Fisher Scientific) represents cellular ATP level and reflects cell viability. To achieve real-time monitoring of live cell viability in an impedance-based instrument system (iCELLigence, ACEA Biosciences) as previously described [17], the TNBC cells were cultured on electronic plates (E-Plates L8, ACEA Biosciences) at a density of 4 × 10^4^ cells/400 μL complete medium for 96 h. The impedance was consistently monitored every 15 min.

### 2.3. Cell Proliferation Assays

To assess the long-term effects of siRNA against DYRK1B or inhibitor AZ-191 (R&D, 5232) on cell viability, TNBC cells were reversely transfected with siRNA in 12-well plates (5 × 10^3^ cells/well) or cultured overnight for AZ-191 treatment. The culture media was refreshed every 3 d without adding new siRNA or inhibitor for around 2 w until colony formation was achieved. The colonies were further fixed, stained with crystal violet, and counted to determine the cytotoxic effects of cells. To precisely determine the effects of DYRK1B on cell cycle progression, the transfected cells were added with BrdU (10 µM) 1 h before harvesting as reported previously (2014 ATG4B, 2017 CPB). The cells were then fixed and the BrdU incorporated cells were determined by anti-BrdU antibody conjugated with FITC and 7-aminoactinomycin D (7AAD). The proportion of cell cycle progression was determined by a FACScan (Becton Dickinson) and analyzed by FlowJo (Tree Star).

### 2.4. Spheroid Cell Culture

The TNBC cells were cultured in NanoCulture plates (2 × 10^4^/well, SCIVAX Corporation, Kanagawa, Japan) and 96-well ultralow attachment plates (4 × 10^3^/well, Costar^®^, New York, NY, USA). Cells transfected with 10 nM scramble or siRNA against DYRK1B were cultured for 7 d to observe tumorsphere of the control and silenced tumorsphere under microscope. Tumorsphere viability was evaluated by 3D CellTiter Glo (G9681, Promega) or Calcein AM (Green)/Ethidium homodimer-1 (EthD-1, Red) (LIVE/DEAD^®^ Viability/Cytotoxicity Kit, ThermoFisher Scientific) to differentiate live and dead cells. Tumorsphere viability was evaluated and quantitated by Fluoroskan Ascent FL reader (Thermo Fisher Scientific), as previously described [17].

### 2.5. Cell Mobility Assay

For the wound healing assay, 1 × 10^5^ TNBC cells were cultured in the inserts (IBIDI, Inc., Planegg, Germany) until the cells attach well. The inserts were then taken out for cell migration and the blank area measured between time 0 and end points was considered as migration ability. For invasion assay, the cells were seeded with DMEM medium and 1% FBS in the inserts (8-μm pore, Greiner Bio-One, Stroud, UK) until the cell invaded to another side of the insert. The cells were then stained with 0.25% crystal violet and imaged under a microscope at a magnification of ×200.

### 2.6. Real-Time PCR (RT-PCR)

The transfect cells were harvested for total RNA extraction with TRIzol Reagent (15596-018, Invitrogen). The 1 µg RNA was converted to cDNA by SuperScript II RNase H-Reverse Transcriptase (18064-014, Invitrogen) and the mRNA of each gene was amplified (StepOnePlus^TM^ system, Applied Biosystems) and stained with SYBR Green Master Mix (4385612, Applied Biosystems) for quantification. The primer sequences for control GAPDH, kinase, and their correlated genes will be provided upon request. 

### 2.7. Western Blot

The protein of cells was extracted for immunoblotting as described previously [18,19,20]. Briefly, the cells were harvested with lysis buffer containing protease inhibitor cocktail. The proteins were separated by SDS-PAGE and transferred to nitrocellulose membranes for further immunoblotting with the primary antibodies, including anti-DYRK1B (1:1000, 5672, Cell Signaling Technology), anti-His tag (1:1000, 12698, Cell Signaling Technology), and anti-ACTB (1:2000, A5441, Sigma) at 4 °C overnight, followed by probing with secondary antibody and ECL reagent. The membrane was scanned and analyzed for protein expression as uncropped images in Figure S1 with a BioSpectrum^®^ Imaging System (UVP, Upland, CA, USA). 

### 2.8. Immunohistochemistry and Scoring

Surgically resected tissues obtained from 59 TNBC patients were used to evaluate the correlation of DYRK1B protein with TNBC as the retrospective cohort in this study. Written informed consent was provided by all participants in this study. The study was approved by Institutional Review Board (IRB) of Kaohsiung Veterans General Hospital (No. KSVGH20-CT1-08). Tissue microarray preparation and immunohistochemistry was carried out as previously reported [14,21,22]. Briefly, the protein level of DYRK1B on tissues was probed with anti-DYRK1B (1:100, Ab124960, Abcam, Cambridge, UK), then followed by a horseradish peroxidase-conjugated secondary antibody and Bond Polymer Refine Detection kit (DS9800, Leica Biosystems). Since the staining signal of DYRK1B on tissues is quite homogenous, the scoring is based on the staining intensity, which can be divided into negative (0), weak (1), moderate (2), and strong (3) levels. 

### 2.9. Statistics

For comparison of cell viability between the control and DYRK1B-silenced cells, the nonparametric 2-tailed Student *t* test is used (*p* < 0.05 is considered as significance). The association of gene expression with TNBC patients was analyzed using the TCGA database obtained from the UCSC Xena website (https://xenabrowser.net/datapages/, 1 May 2021). The TNBC patients (178 cases, negative expression of ER, PR, and HER2) is defined from the clinical dataset, which is determined by pathologists according to the guideline made by the American Society of Clinical Oncology (ASCO) and the College of American Pathologists (CAP) [23,24]. Briefly, the expression levels of ER, PR, and HER2 are mainly determined by immunohistochemistry. The finding of <1% of tumor cell nuclei is considered as negative for ER and PR, while membrane staining in <10% of cells is considered as negative for HER2. The gene expression levels of DYRK1B in tissues between the tumors and corresponding tumor adjacent normal (CTAN) were evaluated with Wilcoxon signed-rank test. To separate high or low gene expression in tumor tissues for survival analyses, the ROC cutoff was set for DYRK1B, CCDC97, and ZNF581. Cumulative survival curves were estimated using the Kaplan–Meier method. The crude and adjusted hazard ratios are respectively calculated with univariate and multivariate Cox proportional hazards models. 

## 3. Results

### 3.1. Kinome-Wide siRNA Library Screening for the Kinases Required for Proliferation of TNBC Cells

**Figure 1:**
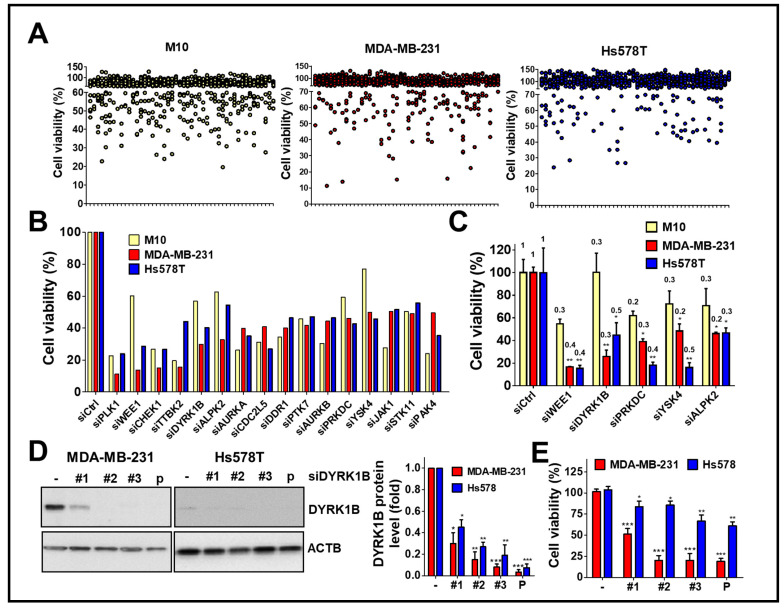
High throughput screening with the kinome siRNA library for kinases essential for cell viability of TNBC cells. (**A**) TNBC cell lines MDA-MB-231 and Hs578T were reversely transfected with arrayed siRNA (10 nM) against kinase gene in 384-well plates for 72 h using normal mammary epithelial cell H184B5F5/M10 (M10) cell line as a counter assay. The cell viability of siRNA transfected cells was determined by Cell-Titer Glo. (**B**) The top 16 gene hits involved in cell viability in TNBC cells were selected after ruling out the same hits from normal H184B5F5/M10 cells. (**C**) Real-time PCR and cell viability were used to determine the knockdown efficiency (fold change compared to cells with control siRNA as labeled on the top of each bar) of genes showing selective effects on cell viability among normal H184B5F5/M10 and TNBC MDA-MB-231 and Hs578T cells. (**D**) The knockdown efficiency of individual siRNA (#1, #2, and #3) and pooled (P) siRNA against DYRK1B in MDA-MB-231 and Hs578T cells was validated by immunoblotting and quantitated in the right panel. (**E**) The effects of these siRNAs on cell viability in MDA-MB-231 and Hs578T cells were further examined. Quantifiable results are indicated as the mean ± SD from three separate experiments. * *p* < 0.05, ** *p* < 0.01, *** *p* < 0.001 vs. control siRNA (siCtrl).

To explore kinases involvement in the viability of TNBC cells, human TNBC cells MDA-MB-231 and Hs578T were screened with a kinome siRNA library, consisting of 709 human kinases and related genes, using normal breast epithelial cells H184B5F5/M10 as a control (Figure 1A). The results show the top 16 gene hits (around 2% of kinases related genes) were initially selected due to the silencing of these genes inhibiting cell proliferation of TNBC cells (Figure 1B). After counter screening with H184B5F5/M10 cells, five kinases including mitosis inhibitor protein kinase WEE1, DYRK1B, DNA-dependent protein kinase catalytic subunit (PRKDC), yeast Sps1/Ste20-related kinase 4 (YSK4), and Alpha protein kinase 2 (ALPK2) were selected for further confirmation in terms of knockdown efficiency and cell viability. (Figure 1C).

**Figure 2:**
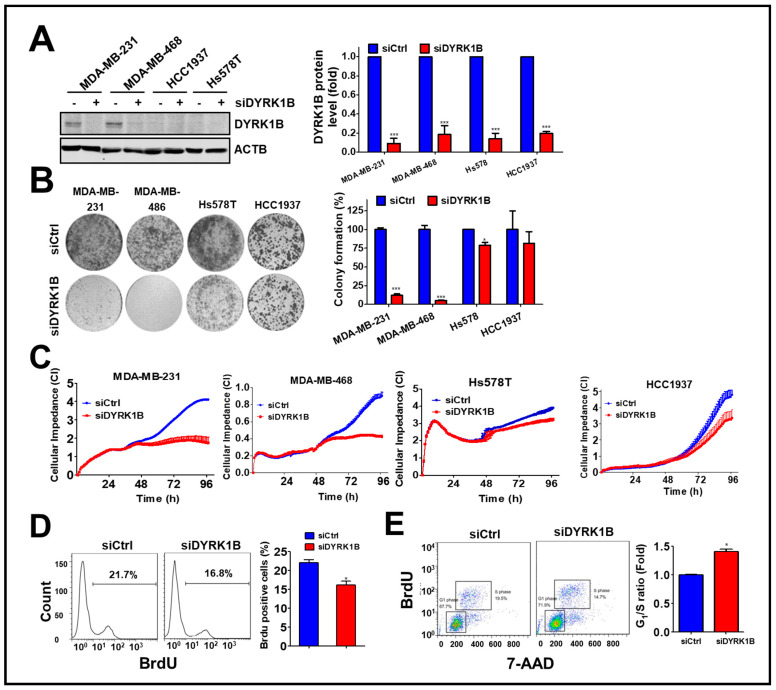
The effects of DYRK1B knockdown on cell proliferation in TNBC cells. (**A**) TNBC cell lines including MDA-MB-231, MDA-MB-468, HCC1937, and Hs578T were administrated with siRNA (10 nM) against the DYRK1B for 72 h. The knockdown efficiency was validated by immunoblotting and quantitated in the right panel. (**B**) The effects of gene silencing on cell proliferation in TNBC cells were determined by clonogenic assay. Colony formation is quantitated in the right panel. (**C**) MDA-MB-231, MDA-MB-468, HCC1937, and Hs578T cells were seeded on electronic plates and transfected with scrambled siRNA or siRNA against DYRK1B (10 nM). The cell proliferation of transfected cells was monitored in real-time using an impedance-based system. (**D**) The effects of DYRK1B knockdown on cell cycle phases of MDA-MB-231 cells were determined by BrdU incorporation assays (left panel). The BrdU-positive cells were quantified to express DNA replication in the S phase. (**E**) The ratio of the G_1_/S phase in cells in (**D**) were validated with BrdU/7-AAD staining and analyzed by flow cytometer (tangle, left panel). The ratio of the G_1_/S phase was quantitated with FlowJo software. The results are indicated as the mean ± SD from three individual experiments * *p* < 0.05 or *** *p* < 0.001 vs. non-targeting control siRNA (siCtrl).

Following the silencing of these candidate genes, mRNA levels and cell viability were found to both decrease, suggesting these five kinase genes play a crucial role in the growth of TNBC cells. Among these genes, knockdown of DYRK1B significantly reduced cell viability in MDA-MB-231 (25%) and Hs578T (45%) TNBC cells, but it had no effect on normal H184B5F5/M10 cells (100%). Although silencing the other kinases significantly inhibited cell viability of MDA-MB-231 and Hs578T cells (<47%), the siRNAs also reduced cell viability of normal H184B5F5/M10 cells, ranging 55~72%. These results show DYRK1B had the highest selectivity on cell viability between normal and TNBC cells compared with the differential effects resulting from knockdown of the other candidate genes (Figure 1C). To eliminate off-target effects of siRNA, silencing DYRK1B with three individual siRNA showed knockdown efficiency (Figure 1D). The effects of these siRNA on cell viability were also consistently with the knockdown efficiency (Figure 1E). Moreover, DYRK1B expression was examined in various TNBC cell lines, including MDA-MB-231, MDA-MB-468, HCC1937, and Hs578T cells. MDA-MB-231 and MDA-MB-468 cells showed higher protein expression levels of DYRK1B, whereas HCC1937 and Hs578T cells had much lower expression (Figure 2A). In contrast with DYRK1B-low expressed TNBC cells, deprivation of DYRK1B almost completely suppressed colony formation in high DYRK1B expressed TNBC cells (Figure 2B). Likewise, the TNBC cells were also cultured in an impedance-based system to monitor the cell proliferation in live cells (Figure 2C). Silencing DYRK1B largely decreased cell growth in high DYRK1B expressed TNBC cells (MDA-MB-231 and MDA-MB-468). However, the silencing DYRK1B had little effect on growth inhibition in low DYRK1B expressed TNBC cells (HCC1937 and Hs578T), suggesting DYRK1B is crucial for proliferation of TNBC cells. Moreover, the effects of DYRK1B on proliferation of MDA-MB-231 cells were analyzed by BrdU incorporation assay (Figure 2D). DYRK1B knockdown significantly reduced BrdU incorporation in MDA-MB-231 cells. The DYRK1B silenced cells were further stained with BrdU-7AAD and analyzed for cell cycle progression. The ratio of G_1_/S phases was significantly increased in cells transfected with siRNA against DYRK1B (Figure 2E). These results suggest that DYRK1B may be critical for the cell cycle progression during the G_1_/S phase.

### 3.2. DYRK1B Confers to Spheroid Cell Formation of TNBC Cells

**Figure 3:**
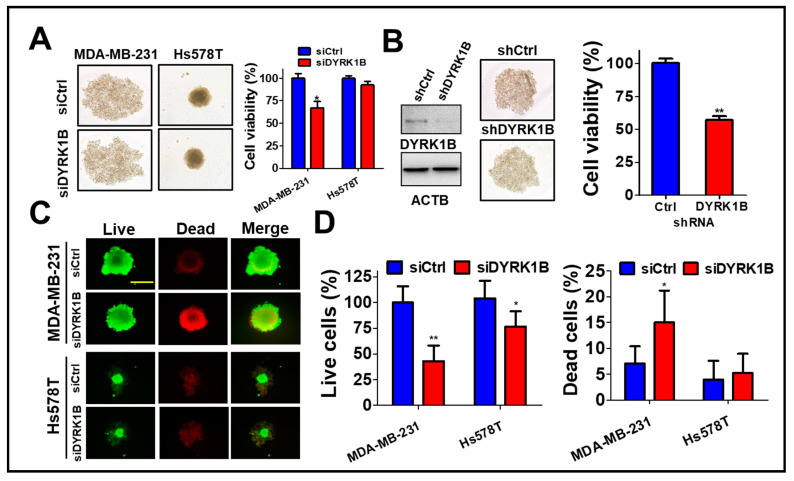
The effects of DYRK1B knockdown on spheroid formation of TNBC cells. (**A**) TNBC MDA-MB-231 cells were cultured in nanoparticle plates in the presence of scrambled siRNA or siRNA against DYRK1B for spheroid cell formation. The viability of tumorspheres was measured with 3D Cell-Titer Glo (**B**) The MDA-MB-231 cells expressing scrambled shRNA or shRNA against DYRK1B were cultured on a nanoparticle plate and tumorsphere viability was determined with 3D Cell-Titer Glo. (**C**) MDA-MB-231 and Hs578T cells were transfected with scrambled siRNA or siRNA against DYRK1B in an ultra-low attachment plate and the viable or dead cells were examined by Live (Green)/Dead (Red) cytotoxicity assay. The representative results were shown. (**D**) The live and dead cell population was measured according to fluorescence intensity and quantitated. The quantifiable results are labeled as the mean ± SD from three individual experiments. * *p* < 0.05 or ** *p* < 0.01 vs. control siRNA (siCtrl).

To assess the role of DYRK1B in the tumorsphere, MDA-MB-231 cells were cultured on ultra-low attachment plates and transfected with scrambled siRNA or siRNA against DYRK1B (Figure 3A). The tumorsphere viability was decreased in DYRK1B-silenced TNBC, particular in MDA-MB-231 cells, even though the size of tumorsphere was not altered after knockdown of DYRK1B (Figure 3A). Likewise, MDA-MB-231 stably harbored shRNA against DYRK1B diminished DYRK1B expression and tumorsphere viability compared with the scrambled cells (Figure 3B), suggesting DYRK1B is necessary for supporting anchorage independent cancer cell growth, but not for forming spheres. siRNA transfected MDA-MB-231 or Hs578T cells were also cultured on an ultra-low attachment plate for tumorsphere formation (Figure 3C). Compared to low DYRK1B expressed Hs578T cells, high DYRK1B expressed MDA-MB-231 cells were easier to form tumorsphere. The results of LIVE/DEAD assay for the tumorsphere showed that the ablation of DYRK1B significantly reduced the number of live cells in TNBC cells, particularly in high DYRK1B expressed MDA-MB-231 cells. Also, the number of dead cells increased only in MDA-MB-231 cells, not in Hs578T cells (Figure 3D), suggesting that DYRK1B might promote proliferation and survival in the TNBC cells.

### 3.3. DYRK1B Kinase Activity Is Required for Malignancy of TNBC Cells

**Figure 4:**
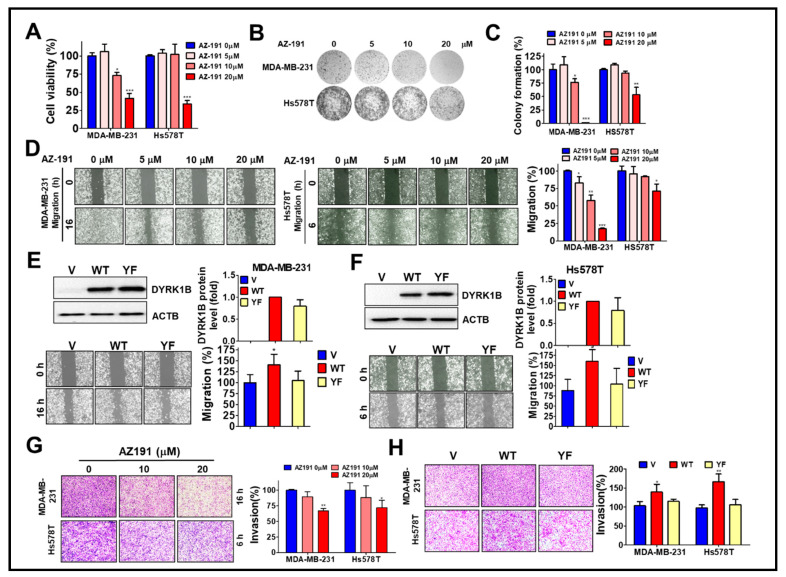
The involvement of DYRK1B kinase activity in cell proliferation and metastatic characteristics of TNBC cells. (**A**) MDA-MB-231 and Hs578T cells were treated with 5, 10, or 20 µM DYRK1B inhibitor AZ-191 for 24 h. The cell viability was determined with Cell-Titer Glo. (**B**) The TNBC cells were exposed to AZ-191 overnight and the medium was refreshed to remove excessive inhibitor and culture until colony formation. The colonies were fixed and stained with crystal violet. (**C**) The colony number was counted and quantitated. The quantitative results are expressed as the mean ± SEM from three independent experiments. (**D**) The MDA-MB-231 and Hs578T cells were treated with AZ-191 to examine cell migration ability. The migration distance of cells was measured, and the quantitative results were shown. (**E**) The MDA-MB-231 and (**F**) Hs578T cells were transfected with vector or vector encoding DYRK1B wild-type (WT) or catalytic mutant (YF) to examine protein expression in the upper panel and migration in the lower panel. Migratory ability is quantitated in the right panel. (**G**) MDA-MB-231 and Hs578T cells were seeded on Matrigel-coated Transwell filters and treated with AZ-191 to inspect cell invasion. (**H**) The cells as panel E and F were examined for their ability in invasion. The invasive effects of DYRK1B activity in TNBC cells are quantitated and shown. The results are indicated as the mean ± SD from three individual experiments. * *p* < 0.05, ** *p* < 0.01, and *** *p* < 0.001 vs. DMSO (0) or vector (V).

To determine whether the kinase activity of DYRK1B plays a role in cell growth and malignancy of TNBC, high DYRK1B expressed MDA-MB-231 and low DYRK1B expressed Hs578T cells were treated with DYRK1B inhibitor AZ-191 for cell viability assay (Figure 4A). AZ-191 significantly decreased the viability of MDA-MB-231 and Hs578T cells in a dose-dependent manner. Furthermore, 20 µM of AZ-191 largely diminished the colony formation of both MDA-MB-231 and Hs578T cells (Figure 4B). Of note, the suppressive effects of AZ-191 on colony formation of MDA-MB-231 cells was relatively obvious compared to that in Hs578T cells (Figure 4B,C). Similarly, AZ-191 inhibited migration and invasion in MDA-MB-231 cells, whereas AZ-191 had a marginal effect on migration and invasion of Hs578T cells (Figure 4D,G). Nevertheless, IC_50_ of AZ-191 for DYRK1B and DYRK1A is 17 nM and 88 nM, respectively [25]. Low dose of AZ-191 (<3 µM) is sufficient to suppress cell proliferation and/or migration in liposarcoma cells and hepatocellular carcinoma cells [26,27]. We also observed that 20 µM of AZ-191 induced cell death and G_2_/M phase arrest in MDA-MB-231 cells, whereas 5 or 10 µM of AZ-191 did not have any effects on cell death or cell cycle arrest. Thus, it requires much higher concentration to observe the inhibitory effects in cells compared to that in cell-free (in vitro) assay, likely due to specificity, permeability, metabolism, and efflux efficiency of AZ-191 in different cells. There is little doubt if the inhibitory effects of AZ-191 in TNBC cells was DYRK1B-dependent. To further determine whether kinase activity of DYRK1B is required for migration, the MDA-MB-231 and Hs578T cells were transfected with the expression vector encoding human gene of DYRK1B wild-type (WT) or catalytic mutant (YF). TNBC cells ectopically expressing wild-type DYRK1B migrated and invaded faster than the TNBC cells harboring vector only or catalytic mutant (YF) (Figure 4E,F,H). Although the efficacy of AZ191 and transfection in each cell line might be variable, these results imply that the kinase activity of DYRK1B might be partially involved in cancer cell proliferation and metastatic characteristics.

### 3.4. The Correlation of DYRK1B Expression with Cancer Development and Survival in TNBC Patients

**Figure 5:**
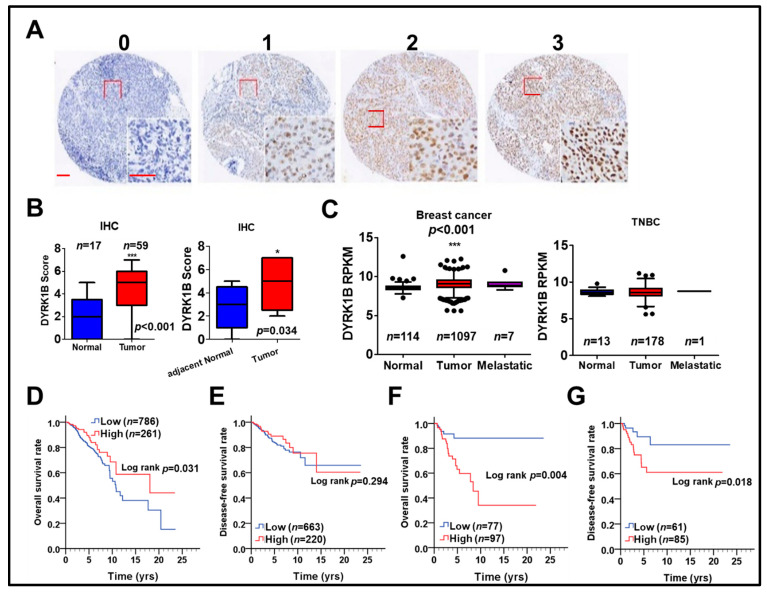
The relationship of DYRK1B expression in tumor tissues and survival of breast cancer patients. (**A**) DYRK1B protein level on tissues of TNBC patients was categorized into three grades, including no (0), weak (1), moderate (2), and strong (3) expression, according to the staining results of immunohistochemistry. Scale bar: 100 µm. (**B**) The differential protein level of DYRK1B on tumor tissues and normal (left panel) or adjacent normal (right panel, *n* = 9) tissues of TNBC patients was quantified. * *p* < 0.05, *** *p* < 0.001 vs. normal tissues in TNBC patients. (**C**) Gene expression of DYRK1B in normal and tumor tissues in breast cancer patients (left panel) TNBC patients (right panel) was analyzed according to TCGA database. The *y*-axis is shown as reads per kilobase of transcript per million mapped reads (RPKM) to represent transcription level of the DYRK1B gene. *** *p* < 0.001 vs. tumor adjacent normal tissues in TNBC patients. The association of DYRK1B gene in (**D**) overall survival and (**E**) disease-free survival in breast cancer patients was analyzed by Kaplan–Meier plots. The association of DYRK1B expression with (**F**) overall survival (**G**) disease-free survival of TNBC patients was further evaluated.

To examine the association of DYRK1B with cancer development and survival of breast cancer patients, protein and gene levels of DYRK1B were used to evaluate the association with our cohort (Figure 5A,B) and The Cancer Genome Atlas (TCGA) database (Figure 5C–G), respectively. The protein level of DYRK1B was significantly elevated on tumor tissues compared to that in normal (*p* < 0.001) or adjacent normal tissues (*p* = 0.034) of TNBC patients (Figure 5A,B). DYRK1B gene expression was substantially higher in tumor tissues compared with that in normal tissues of all breast cancer patients (Figure 5C; *p* < 0.001). Among all breast cancer patients, DYRK1B expression in TNBC patients (negative expression of ER, PR, and HER2) was relative lower than that in the other subtypes (Table S1). Also, DYRK1B expression was significantly elevated in tumor tissues of any other subtypes except TNBC (Table S2). Moreover, there was no significant difference between normal and tumor tissues in TNBC patients (Figure 5C). High expression of DYRK1B was considerably associated with better overall survival in all types of breast cancer patients (Figure 5D), but it had no significant correlation with disease-free survival (Figure 5E). However, TNBC patients with high expression of DYRK1B had significantly poor overall (Figure 5F) and disease-free survival (Figure 5G). 

To further specify the correlation of DYRK1B expression with prognosis in different subtypes of breast cancer, including ER-positive, PR-positive, HER2-positive, and TNBC, the relationship of DYRK1B expression and overall survival or disease-free survival was analyzed in breast cancer patients (Table 1). Univariate analysis results showed that high DYRK1B expression was substantially associated with worse overall survival only in TNBC patients (Table 1; crude hazard ratio (CHR) = 3.22, *p* = 0.025). Thus, DYRK1B may confers to tumor progression of TNBC and result in worse survival. Nevertheless, high DYRK1B expression was markedly associated with poor disease-free survival in TNBC, ER-, and PR-positive patients (Table 1; TNBC, CHR = 4.22, *p* = 0.026; ER, CHR = 1.70, *p* = 0.045; PR, CHR = 2.18, *p* = 0.019). These results imply that DYRK1B might be involved in recurrent of different subtypes of breast cancer except TNBC.

The Kaplan–Meier survival curve also indicated that high DYRK1B expression was significantly related with poor overall and disease-free survival in TNBC patients (Figure 5F, *p* = 0.004, Figure 5G, *p*= 0.018). Nevertheless, multivariate analysis after adjustment with AJCC pathological stage showed that high DYRK1B was associated with significantly shorter disease-free survival in PR positive breast cancer patients (Table 1; adjusted hazard ratio (AHR) = 2.10, *p* = 0.027). The connection of DYRK1B expression with prognosis in certain clinicopathological groups of TNBC was further investigated. Interestingly, high DYRK1B expression was correlated to poor overall survival in TNBC patients without radiation therapy (Table S3, CHR = 10.30, *p* = 0.029). However, there was no significant correlation with prognosis according to other clinicopathological factors in terms of either overall survival or disease-free survival of TNBC patients. 

### 3.5. Co-Expression of DYRK1B and CCDC97 or ZNF581 Correlates with Prognosis of TNBC Patients

**Figure 6:**
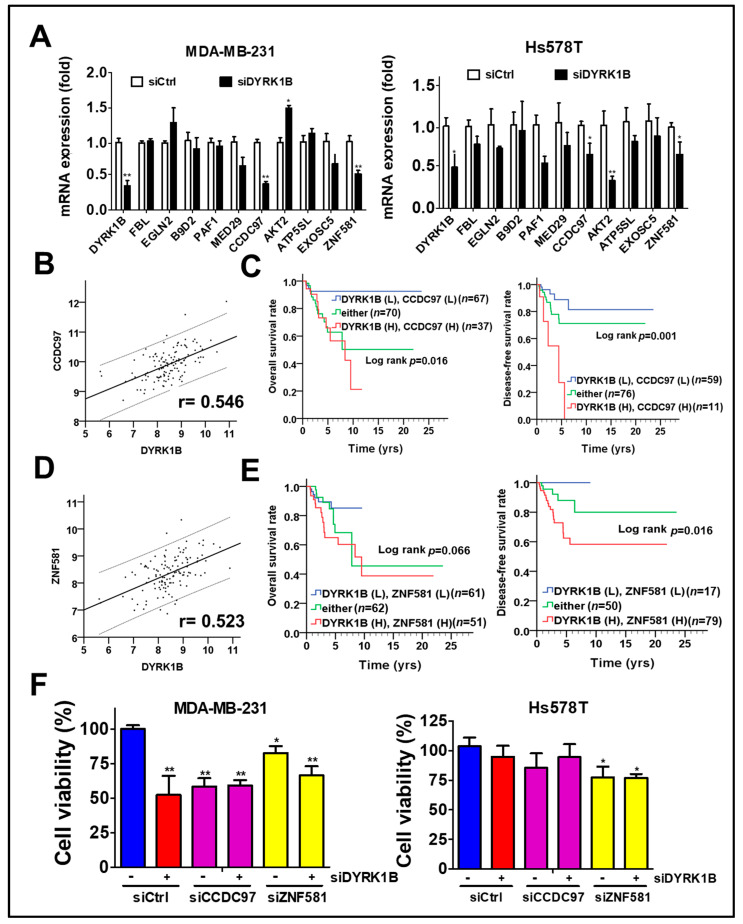
The association of DYRK1B and potential regulators with prognosis in TNBC patients. (**A**) The mRNA levels of the 10 genes most highly correlated with DYRK1B expression were examined in DYRK1B-silenced TNBC cells, including MDA-MB-231 and Hs578T cells. CCDC97 and ZNF581 mRNA levels are consistent with DYRK1B in TNBC cells. (**B**) The expression correlation of DYRK1B and CCDC97 and (**D**) ZNF581 was analyzed using Pearson χ2 test. (**C**) The relationship of co-expression of DYRK1B and CCDC97 or (**E**) ZNF581 with prognosis was inspected using Kaplan–Meier analysis. The associations of co-expression with overall survival and disease-free survival in TNBC patients are, respectively, shown in left panels and right panels. (**F**) TNBC cells were silenced with DYRK1B in the absence or presence of siRNA against CCDC97 or ZNF581 for 72 h. The cell viability of transfected cells was determined by Cell-Titer Glo. The results are indicated as the mean ± SD from three individual experiments * *p* < 0.05, ** *p* < 0.01 vs. scramble siRNA (siCtrl).

To determine whether any potential DYRK1B-modulated factors might be associated with unfavorable prognosis in TNBC patients, we explored the correlation of DYRK1B with all human genes in the TNBC dataset obtained from TCGA database, which contains over 20,000 genes. Top 10 DYRK1B-correlated genes were examined for their expression in DYRK1B-silenced TNBC cells. The expression of coiled-coil domain-containing protein 97 (CCDC97) and zinc finger protein 581 (ZNF581) was consistently decreased in DYRK1B-silenced MDA-MB-231 cells (Figure 6A). Furthermore, the clinical relevance of these genes in TNBC was evaluated with TCGA database. Pearson correlation analysis showed that CCDC97 and ZNF581 were highly correlated with DYRK1B expression in TNBC patients (Figure 6B, CCDC97: r = 0.546, *p* < 0.001; Figure 6D, ZNF581: r = 0.523, *p* < 0.001). In addition, the survival curve revealed that high co-expression of DYRK1B and CCDC97 was correlated with worse overall survival (Figure 6C, *p* = 0.016) and disease-free survival (*p* = 0.001). High co-expression of DYRK1B and ZNF581 was only correlated with worse disease-free survival (Figure 6E, *p* = 0.016) The TNBC cells were further silenced with siRNA against DYRK1B along with siRNA against CCDC97 or ZNF581 (Figure 6F). The cell viability was significantly decreased in DYRK1B or CCDC97/ZNF581-silenced MDA-MB-231, while knockdown of both DYRK1B and CCDC97 or ZNF581 had no additional effects compared to the cells with siRNA against single gene (Figure 6F). In contrast, knockdown of DYRK1B or CCDC97 had little or no effect in Hs578T cells. Ablation of ZNF581 had modest effects in Hs578T cells. Similar to MDA-MB-231, combinational knockdown had no additional effects compared with signal gene knockdown (Figure 6F).

After adjustment with AJCC pathological stage in the multivariate model, high co-expression of DYRK1B and CCDC97 had significant association with shorter overall survival (Table 2; AHR = 4.52, CI = 1.42–14.42, *p* = 0.011) and disease-free survival (AHR = 3.26, CI = 1.03–10.36, *p* = 0.045). Similarly, high co-expression of DYRK1B and ZNF581 was substantially associated with unfavorable overall survival (Table 3; AHR = 2.88, CI = 1.11–7.46, *p* = 0.029) and disease-free survival (Table 3; AHR = 3.36, CI = 1.05–10.72, *p* = 0.041). These results suggest that DYRK1B might coordinate with CCDC97 or ZNF581 for tumor malignancy in TNBC patients. 

## 4. Discussion

There is no specific therapeutic target for TNBC treatment. Conventional chemotherapy for TNBC patients results in serious side effects. Thus, therapeutic targets for future treatments of TNBC patients are urgently needed. Kinases play crucial roles in tumorigenesis, tumor malignancy, and survival in stressed microenvironments. Many kinases have been used as therapeutic targets for cancer treatments. However, little is known about the role of kinome in TNBC therapy. Herein, a kinome siRNA library was used to scan for potential kinases required for cell viability of TNBC cells, with the following findings: first, DYRK1B knockdown selectively blocked cell viability of TNBC cells, particularly in DYRK highly expressed TNBC cells; second, removal of DYRK1B arrested cell cycle progression at G_1_ phase and reduced tumorsphere formation; third, DYRK1B inhibitor or overexpression of DYRK1B catalytic mutant diminished proliferation, migration and invasion of TNBC cells; fourth, tumor tissues with high DYRK1B expression levels were associated with poor prognosis for TNBC patients; and, fifth, through TNBC whole genome data analysis for DYRK1B-correlated gene in TCGA database, DYRK1B was positively correlated with CCDC97 and ZNF581. In addition, co-expression of DYRK1B and CCDC97 or ZNF581 was associated with unfavorable survival rates in TNBC patients. These results suggest that DYRK1B might be a new target for TNBC therapy. 

There are five kinase members in the DYRK family of the human genome, including DYRK1A, DYRK1B, DYRK2, DYRK3, and DYRK4 [28]. All DYRK family members contain a DYRK homology (DH) domain and catalytic domain, while DYRK1A and DYRK1B share the highest similarity in the sequence of catalytic domains. In addition, DYRK1A and DYRK1B comprise the N-terminal nuclear localization sequence (NLS) and C-terminal proline, glutamate, serine, and threonine (PEST) degradation sequence. DYRK1A and DYRK1B can phosphorylate cyclin D1 (CCND1) and p27^kip1^ to maintain cells in a quiescent G_0_ state [29]. Moreover, DYRK1A and DYRK1B turn on antioxidant gene expression to maintain cellular reactive oxygen species (ROS) levels. These findings suggest that DYRK1A and DYRK1B may have similar physiological functions and can mutually compensate for their biological functions. However, our screening results showed that only silencing DYRK1B has selective effects on cancer cell suppression in TNBC cells compared to normal breast M10 cells. In contrast, silencing DYRK1A had no effect on M10 and MDA-MB-231 cells (remain 102% and 100% cell viability in M10 and MDA-MB-231, respectively). Previous reports also indicate that ablation of DYRK1B inhibits colony formation in pancreatic cancer cells, whereas it had little or no effect in DYRK1A-depleted pancreatic cancer cells. Together with our results, these findings imply that DYRK1B might have a different route to regulate cell proliferation in cancer cells.

As previously mentioned, DYRK1B serves as a cell cycle check point to block G_0_ to S phase. Overexpression of DYRK1B causes cell cycle arrest, while silencing DYRK1B leads to cells entering S phase in fibroblast and myoblast cells [30]. Similar effects on cell cycle arrest are observed in serval cancer cells such as ovarian cancer and colorectal cancer cells [29]. Nevertheless, depletion of DYRK1B decreased colony formation of pancreatic cancer and lung cancer cells [31,32]. Our present results indicate that DYRKB expression is elevated in TNBC patients. Knockdown of DYRK1B selectively inhibited growth of TNBC cells, but not of normal breast M10 cells. Pharmacological and genetic inhibition of DYRK1B also blocked cell proliferation, particularly in DYRK1B highly expressed TNBC cells. These results suggest that the role of DYRK1B on cell cycle regulation might vary in different cancer cells. In addition, ablation of DYRK1B increases ROS production to over the threshold of affordable ROS levels and enhances cell death in cancer cells when exposed to anticancer drugs [33,34], indicating that knockdown or inactivation of DYRK1B may have a synergistic effect with chemotherapeutic drugs on TNBC cells. In line with previous findings, our current study shows that high expression of DYRK1B is correlated with disease-free survival in TNBC patients, supporting the notion that DYRK1B might serve as a drug resistant gene and results in recurrence of cancer cells. 

Regarding the mechanisms of DYRK1B on cancer progression, silencing DYRK1B consistently reduced two rare genes, CCDC97 and ZNF581, the physiological functions of which have not been previously reported. However, CCDC97 and ZNF581 contain either a coiled coil domain or zinger finger domain, which are common domains in transcriptional regulators. More interestingly, DYRK1B can enhance a zinger finger protein, glioma associated transcription factor (GLI1) [35], to increase canonical Hedgehog (Hh) signaling, which is essential for cancer development and stemness [36]. We found that DYRK1B was positively correlated with CCDC97 and ZNF581 and co-expression of DYRK1B/these proteins was associated with shorter overall survival and disease-free survival. Nevertheless, the detailed mechanisms by which CCDC97 and ZNF581 are involved in DYRK1B-promoted proliferation and metastatic characteristics in TNBC cells still require further research.

Of note, though DYRK1B expression was significantly elevated in breast cancer patients, its expression had no significant difference between tumor tissues and CTAN tissues in TNBC patients. In our cell line studies, MDA-MB-231 and MDA-MB468 had high expression of DYRK1B, whereas HCC1937 and Hs578T had very little DYRK1B expression. These results imply that DYRK1B expression may result from various genetic backgrounds, not subtypes of breast cancer. For example: MDA-MB-231 and MDA-MB468 have wild type of BRCA1, whereas HCC1937 has mutated BRCA1 [37], Thus, the protein levels of DYRK1B in tumor tissues and the regulation mechanisms of DYRK1B in tumors need further empirical verification. Nevertheless, our current results might suggest DYRK1B as a potential therapeutic target for DYRK1B-positive TNBC patients. 

## 5. Conclusions

DYRK1B is one of the most selective genes required for proliferation and metastatic characteristics of TNBC cells among kinome genes. TNBC patients with high expression of DYRK1B were associated with worse survival, suggesting that DYRK1B could be a theranostic target for TNBC.

## Figures and Tables

**Table 1:** The impact of DYRK1B expression levels on survival in breast cancer patients with different molecular subtypes.

Variable		No. (%)	CHR (95% CI)	*p* Value *	AHR (95% CI)	*p* Value ^†^
Overall survival						
TNBC	Low	97 (55.7)	1		1	
	High	77 (44.3)	3.42 (1.40–8.39)	**0.007**	2.45 (0.98–6.11)	0.055
ER	Low	520 (65.4)	1		1	
	High	275 (34.6)	0.93 (0.62–1.40)	0.741	0.80 (0.52–1.23)	0.303
PR	Low	444 (64.3)	1		1	
	High	246 (35.7)	0.85 (0.55–1.32)	0.475	0.74 (0.47–1.19)	0.213
HER2	Low	35 (31.5)	1		1	
	High	76 (68.5)	1.65 (0.45–6.00)	0.449	2.17 (0.58–8.15)	0.25
Disease-free survival						
TNBC	Low	85 (58.2)	1		1	
	High	61 (41.8)	3.11 (1.16–8.37)	**0.024**	2.03 (0.74–5.57)	0.167
ER	Low	322 (48.1)	1		1	
	High	348 (51.9)	1.70 (1.01–2.87)	**0.045**	1.54 (0.91–2.62)	0.11
PR	Low	247 (42.3)	1		1	
	High	337 (57.7)	2.18 (1.14–4.17)	**0.019**	2.10 (1.09–4.04)	**0.027**
HER2	Low	61 (70.1)	1		1	
	High	26 (29.9)	2.63 (0.53–13.08)	0.237	3.89 (0.65–23.40)	0.138

Abbreviations: TNBC, Triple-negative breast cancer; CHR, crude hazard ratio; CI, confidence interval; AHR, adjusted hazard ratio; AJCC, American Joint Committee on Cancer; and RT, radiotherapy. * *p* Values were estimated by Cox’s regression. ^†^ *p* Values were adjusted for AJCC pathological stage (stage III + IV vs. stage I + II) by multivariate Cox’s regression. Bold: *p* < 0.05.

**Table 2:** The impact of DYRK1B/CCDC97 co-expression on overall and disease-free survival in TNBC patients.

Variable	ROC Cutoff	No. (%)	CHR (95% CI)	*p* Value *	AHR (95% CI)	*p* Value ^†^
Overall survival						
DRYK1B	Low	77 (44.3)	1.00		1.00	
	High	97 (55.7)	3.42 (1.40–8.39)	**0.007**	2.45 (0.98–6.11)	0.055
CCDC97	Low	127 (73.0)	1.00		1.00	
	High	47 (27.0)	1.65 (0.79–3.43)	0.179	0.97 (0.44–2.12)	0.935
DRYK1B (L) CCDC97(L)		67 (38.5)	1.00		1.00	
Either		70 (40.2)	1.73 (0.84–3.55)	0.136	3.89 (1.30–11.65)	**0.015**
DRYK1B (H) CCDC97(H)		37 (21.3)	1.84 (0.86–3.94)	0.117	4.52 (1.42–14.42)	**0.011**
Disease-free survival						
DRYK1B	Low	61 (41.8)	1.00		1.00	
	High	85 (58.2)	3.11 (1.16–8.37)	**0.024**	2.03 (0.74–5.57)	0.167
CCDC97	Low	133 (91.1)	1.00		1.00	
	High	13 (8.9)	3.02 (1.13–8.10)	**0.028**	1.11 (0.40–3.11)	0.842
DRYK1B (L) CCDC97(L)		59 (40.4)	1.00		1.00	
Either		76 (52.1)	1.26 (0.56–2.81)	0.574	2.11 (0.74–6.01)	0.161
DRYK1B (H) CCDC97(H)		**11 (7.5)**	**2.05 (0.84–4.98)**	**0.114**	**3.26 (1.03–10.36)**	**0.045**

Abbreviations: TNBC, Triple-negative breast carcinoma; CHR, crude hazard ratio; CI, confidence interval; AHR, adjusted hazard ratio; AJCC, American Joint Committee on Cancer; and RT, radiotherapy. * *p* values were estimated by Cox’s regression. ^†^ *p* values were adjusted for AJCC pathological stage (stage III + IV vs. stage I + II) by multivariate Cox’s regression. Bold: *p* < 0.05.

**Table 3:** The impact of DYRK1B/ZNF581 co-expression on overall and disease-free survival in TNBC patients.

Variable	ROC	No. (%)	CHR (95% CI)	*p* Value *	AHR (95% CI)	*p* Value ^†^
Overall survival						
DRYK1B	Low	77 (44.3)	1.00		1.00	
	High	97 (55.7)	3.42 (1.40–8.39)	**0.007**	2.45 (0.98–6.11)	0.055
ZNF581	Low	107 (61.5)	1.00		1.00	
	High	67 (38.5)	1.41 (0.68–2.92)	0.351	1.28 (0.61–2.68)	0.513
DRYK1B (L) ZNF581(L)		61 (35.1)	1.00		1.00	
Either		62 (35.6)	0.90 (0.41–1.99)	0.802	1.68 (0.60–4.73)	0.328
DRYK1B (H) ZNF581(H)		51 (29.3)	2.19 (1.06–4.52)	**0.034**	2.88 (1.11–7.46)	**0.029**
Disease-free survival						
DRYK1B	Low	36 (24.7)	1.00		1.00	
	High	110 (75.3)	3.11 (1.16–8.37)	**0.024**	2.03 (0.74–5.57)	0.167
ZNF581	Low	23 (15.8)	1.00		1.00	
	High	123 (84.2)	26.27 (0.23–3032.62)	0.177	201339.08 (0.00–7.239× 10^280^)	0.970
DRYK1B (L) ZNF581(L)		17 (11.6)	1.00		1.00	
Either		50 (34.2)	1.13 (0.50–2.56)	0.768	2.27 (0.71–7.27)	0.167
DRYK1B (H) ZNF581(H)		**79 (54.1)**	**2.02 (0.90–4.55)**	**0.090**	**3.36 (1.05–10.72)**	**0.041**

Abbreviations: TNBC, Triple-negative breast carcinoma; CHR, crude hazard ratio; CI, confidence interval; AHR, adjusted hazard ratio; AJCC, American Joint Committee on Cancer; and RT, radiotherapy. * *p* values were estimated by Cox’s regression. ^†^ *p* values were adjusted for AJCC pathological stage (stage III + IV vs. stage I + II) by multivariate Cox’s regression. Bold: *p* < 0.05.

## Data Availability

The raw data for gene expression in breast cancer patients can be downloaded from TCGA database in UCSC Xena website (https://xenabrowser.net/datapages/, 1 May 2021). The analyzed results for DYRK1B-correlated genes are available online https://drive.google.com/file/d/14ohOL-QJ12mW7cj9Vz7SRYrrxG-9UX3c/view?usp=sharing, 1 May 2021.

## Supplementary Materials

The following are available online at https://www.mdpi.com/article/10.3390/cancers13225779/s1, Table S1: The comparisons of DYRK1B expression with different tissues; Table S2: The DYRK1B expression in tumor tissues and adjacent normal tissues in the three different subtypes of breast cancer. Table S3: Impact of DYRK1B expression levels on overall survival by the different demographic and clinicopathologic factors with TNBC. Figure S1: Uncropped Western blot images.
