# Supplementary material for: Kinome-Wide siRNA Screening Identifies DYRK1B as a Potential Therapeutic Target for Triple-Negative Breast Cancer Cells
Source: Cancers (Basel). 2021 Nov 18;13(22):5779. doi: 10.3390/cancers13225779 (PMC8616396; doi:10.3390/cancers13225779)
Supplement: Supplementary file 1 [file cancers-13-05779-s001.zip › cancers-1422915-supplementary/Table S1-3.pdf]

Table S1. The comparisons of DYRK1B expression with different tissues

Variables	TNBC		ER+		PR+		HER2+		*p*-value*
	Mean±SD	Median	Mean±SD	Median	Mean±SD	Median	Mean±SD	Median	
	(n=178)		(n=809)		(n=699)		(n=166)		
	8.59±0.88[abc]	8.5961	9.20±0.63[ad]	9.2223	9.22±0.63[be]	9.2337	8.91±0.92[cde]	8.8772	<0.001

Abbreviations: TNBC, triple-negative breast carcinoma ; SD, standard deviation.
**p values were estimated by Kruskal-Wallis one-way ANOVA test.*

[a]p<0.001; [b]p<0.001; [c]p=0.0014; [d]p<0.001; [e]p<0.001.

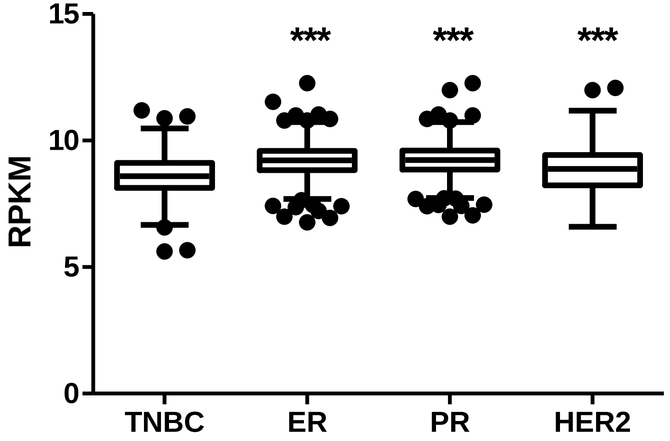

TNBC vs. ER+, p<0.001; TNBC vs. PR+, p<0.001;TNBC vs. HER2+, p=0.00144;

Table S2. The DYRK1B expression in tumor tissues and adjacent normal tissues in the three different subtypes of breast cancer

Variables	Tumor adjacent normal		Tumor		Melastatic		*p*-value
	Mean±SD	Median	Mean±SD	Median	Mean±SD	Median	
	(n=13)		(n=178)		(n=1)		
TNBC	8.67±0.46	8.5871	8.59±0.88	8.5961	8.7507		0.609*
	(n=79)		(n=809)		(n=6)		
ER (+)	8.64±0.55[ab]	8.6020	9.20±0.63[a]	9.2223	9.18±0.86[b]	8.9995	**<0.001**§
	(n=69)		(n=699)		(n=6)		
PR (+)	8.62±0.61[cd]	8.6016	9.22±0.63[c]	9.2337	9.18±0.86[d]	8.9995	**<0.001**§
	(n=21)		(n=166)		(n=1)		
HER2 (+)	8.62±0.34	8.6360	8.91±0.92	8.8772	10.7909		**0.005***

Abbreviations: TNBC, triple-negative breast carcinoma ; SD, standard deviation.

* *p values were estimated by Student's t-test.*

§ *p values were estimated by Kruskal-Wallis one-way ANOVA test.*

[a]p<0.001; [b]p=0.028; [c]p<0.001; [d]p=0.029.

Table S3. Impact of DYRK1B expression levels on overall survival by the different demographic and clinicopathologic factors with TNBC

Variable	DYRK1B	No. (%)	CHR (95% CI)	*p value**	AHR (95% CI)	*p value*†
Sex						
Female	Low	66 (53.7)	1		1	
	High	57 (46.3)	**3.22 (1.16-8.95)**	**0.025**	2.39 (0.84-6.80)	0.104[a]
Age, yrs						
≦50	Low	21 (46.7)	1		1	
	High	24 (53.3)	3.18 (0.66-15.34)	0.149	2.73 (0.54-13.76)	0.225[a]
＞50	Low	45 (57.7)	1		1	
	High	33 (42.3)	3.09 (0.79-12.07)	0.105	2.65 (0.66-10.70)	0.171[a]
AJCC stage						
I, II	Low	56 (57.1)	1		-	
	High	42 (42.9)	2.75 (0.71-10.67)	0.216	-	-
III, IV	Low	9 (40.9)	1		-	
	High	13 (59.1)	2.26 (0.47-10.94)	0.309	-	-
T classification						
T1, T2	Low	61 (56.5)	1		1	
	High	47 (43.5)	2.14 (0.71-6.40)	0.174	1.62 (0.50-5.20)	0.421 [d]
T3, T4	Low	4 (28.6)	1		1	
	High	10 (71.4)	33.40 (0.01-143737.67)	0.411	41.79 (0.01-248111.88)	0.400 [d]
N classification						
N0	Low	49 (62.8)	1		1	
	High	29 (37.2)	4.35 (0.79-24.70)	0.092	4.22 (0.77-23.19)	0.098 [e]
N1, N2, N3	Low	17 (37.8)	1		1	
	High	28 (62.2)	1.60 (0.43-5.93)	0.483	1.53 (0.41-5.68)	0.524 [e]
Postoperative RT						
No	Low	26 (52.0)	1		1	
	High	24 (48.0)	**10.30 (1.26-83.96)**	**0.029**	6.02 (0.67-53.96)	0.109 [a]
Yes	Low	35 (57.4)	1		1	
	High	26 (42.6)	3.11 (0.28-34.45)	0.355	1.50 (0.13-16.84)	0.745 [a]

Abbreviations: TNBC, Triple-negative breast cancer; CHR, crude hazard ratio; CI, confidence interval; AHR, adjusted hazard ratio; AJCC, American Joint Committee on Cancer; RT, radiotherapy. *p values were estimated by Cox's regression. †p values were estimated by multivariate Cox's regression. [a]Adjusted for AJCC pathological stage (stage III+ IV vs stage I+II). [d]Adjusted for N classification (N1, N2, N3 vs N0). [e]Adjusted for T classification (T3+ T4 vs T1+T2).
