# Supplementary figures and images for: Kinome-Wide siRNA Screening Identifies DYRK1B as a Potential Therapeutic Target for Triple-Negative Breast Cancer Cells
Source: Cancers (Basel). 2021 Nov 18;13(22):5779. doi: 10.3390/cancers13225779 (PMC8616396; doi:10.3390/cancers13225779)

**Fig 1D**

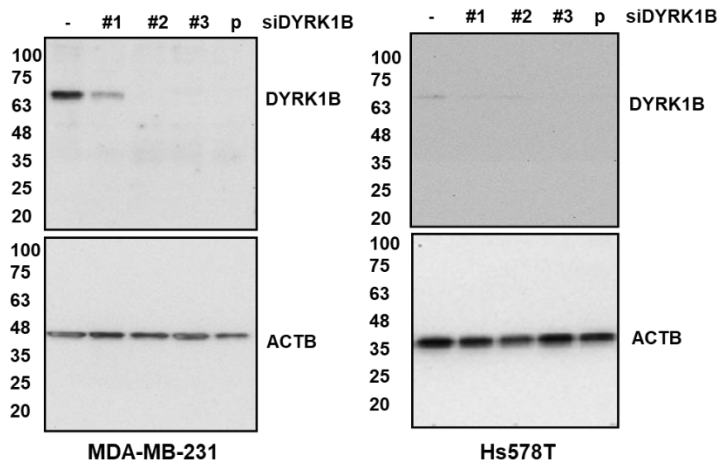

**Fig 2A**

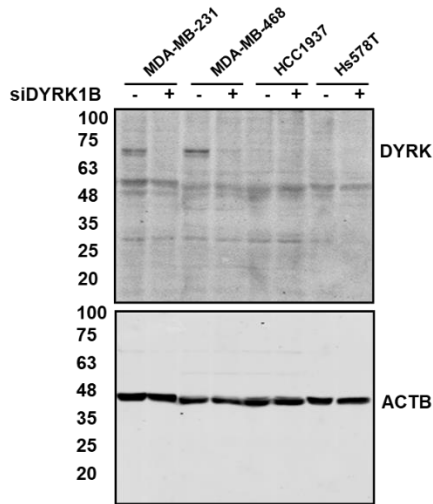

**Fig. 3B**

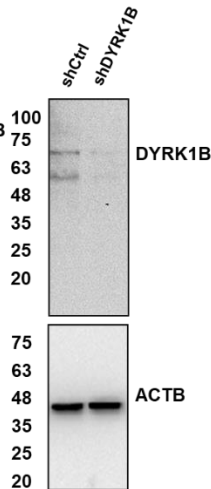

**Fig. 4E**

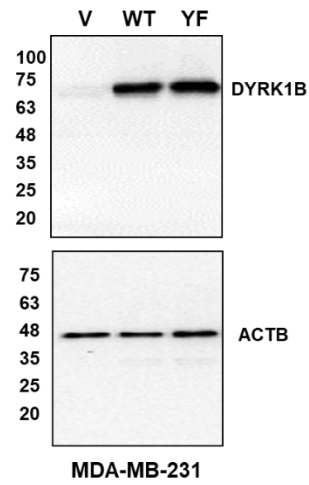

**Fig. 4F**

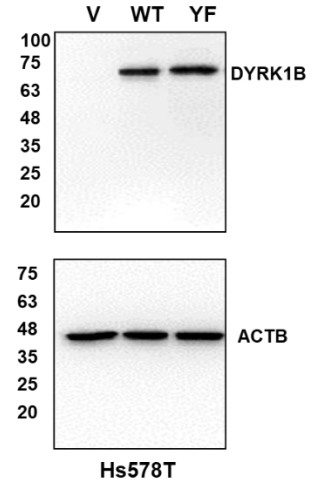

Supplement: Supplementary file 1 [file cancers-13-05779-s001.zip › cancers-1422915-supplementary/WB images.pdf]
